# Supplementary material for: Risk Factors and Outcomes for Multidrug Resistant Pseudomonas aeruginosa Infection in Immunocompromised Patients
Source: Antibiotics (Basel). 2022 Oct 23;11(11):1459. doi: 10.3390/antibiotics11111459 (PMC9686626; doi:10.3390/antibiotics11111459)
Supplement: Supplementary file 1 [file antibiotics-11-01459-s001.zip › antibiotics-1975185-supplementary.pdf]

Supplementary results

Table S1. Phenotypic antibiotic susceptibility patterns in isolates obtained from MDR-PSA cases and non-MDR-PSA controls.

	**MDR-PSA cases** (n = 48)	**Non-MDR-PSA controls** (n = 96)
Susceptible isolates, *n* (%)		
Cefepime	5 (10.4)	95 (98.9)
Ceftazidime	7 (14.6)	94 (97.9)
Ciprofloxacin	3 (6.3)	76 (79.2)
Amikacin	22 (45.8)	94 (97.9)
Piperacillin-tazobactam	3 (6.3)	96 (100.0)
Aztreonam	39 (81.3)	89 (92.7)
Imipenem	3 (6.3)	90 (93.8)
Meropenem	11 (22.9)	92 (95.8)
Colistin	48 (100.0)	96 (100.0)
Carbapenem-resistant isolates, *n* (%)	37 (77.1)	-
XDR phenotype, *n* (%)	6 (12.5)	-
PDR phenotype, *n* (%)	0 (0.0)	-

MDR-PSA: multidrug-resistant *Pseudomonas aeruginosa;* PDR: pandrug-resistant; XDR: extensively drug-resistant
